# Effect of a Moderate-Intensity Aerobic Training on Joint Biomarkers and Functional Adaptations in Rats Subjected to Induced Knee Osteoarthritis

**DOI:** 10.3389/fphys.2019.01168

**Published:** 2019-09-18

**Authors:** Jeanne Brenda Martins, Vanessa Amaral Mendonça, Grazielle Cordeiro Aguiar, Sueli Ferreira da Fonseca, Jousielle Márcia dos Santos, Rosalina Tossige-Gomes, Dirceu de Sousa Melo, Murilo Xavier Oliveira, Hércules Ribeiro Leite, Ana Cristina Resende Camargos, Anderson José Ferreira, Cândido Celso Coimbra, Jacques Poortmans, Vinícius Cunha Oliveira, Sara Barros Silva, Talita Emanuela Domingues, Mário Bernardo-Filho, Ana Cristina Rodrigues Lacerda

**Affiliations:** ^1^Centro Integrado de Pós-Graduação e Pesquisa em Saúde (CIPq-Saúde), Universidade Federal dos Vales do Jequitinhonha e Mucuri (UFVJM), Diamantina, Brazil; ^2^Programa Multicêntrico de Pós-graduação em Ciências Fisiológicas, Sociedade Brasileira de Fisiologia (SBFis), Diamantina, Brazil; ^3^Programa de Pós-Graduação em Reabilitação e Desempenho Funcional (PPGReab), Universidade Federal dos Vales do Jequitinhonha e Mucuri (UFVJM), Diamantina, Brazil; ^4^Instituto de Ciências Biológicas, Universidade Federal de Minas Gerais (UFMG), Belo Horizonte, Brazil; ^5^Programa de Pós-Graduação em Ciências da Reabilitação, Universidade Federal de Minas Gerais, Belo Horizonte, Brazil; ^6^Faculty of Motor Sciences, Université Libre de Bruxelles, Brussels, Belgium; ^7^Departamento de Biofísica e Biometria, Instituto de Biologia Roberto Alcantara Gomes, Universidade do Estado do Rio de Janeiro, Rio de Janeiro, Brazil

**Keywords:** osteoarthritis, aerobic training, exercise, biomarkers, joint function

## Abstract

**Background:**

Knee osteoarthritis (_k_OA) is a common chronic disease that induces changes in redox status and inflammatory biomarkers, cell death, and motor impairment. Aerobic training can be a non-pharmacological alternative to prevent the progression of the disease.

**Objective:**

To evaluate the effects of an 8 weeks moderate-intensity treadmill aerobic training program on redox status and inflammatory biomarkers and motor performance in _k_OA-like changes induced by monosodium iodoacetate (MIA) in rats.

**Methods:**

Twenty-seven rats were randomly divided into three groups: SHAM; induced _k_OA (OA); and induced _k_OA + aerobic training (OAE). Motor performance was evaluated by the number of falls on rotarod test, the total time of displacement and the number of failures on a 100 cm footbridge. Data for cytokines and histology were investigated locally, whereas plasma was used for redox status biomarkers.

**Results:**

The OA group, compared to the SHAM group, increased 1.13 times the total time of displacement, 6.05 times the number of failures, 2.40 times the number of falls. There was also an increase in cytokine and in thiobarbituric acid reactive substances (TBARS) (IL1β: 5.55-fold, TNF: 2.84-fold, IL10: 1.27-fold, IL6: 1.50-fold, TBARS: 1.14-fold), and a reduction of 6.83% in the total antioxidant capacity (FRAP), and of 35% in the number of chondrocytes. The aerobic training improved the motor performance in all joint function tests matching to SHAM scores. Also, it reduced inflammatory biomarkers and TBARS level at values close to those of the SHAM group, with no change in FRAP level. The number of falls was explained by IL1β and TNF (58%), and the number of failures and the total time of displacement were also explained by TNF (29 and 21%, respectively).

**Conclusion:**

All findings indicate the efficacy of moderate-intensity aerobic training to regulate inflammatory biomarkers associated with improved motor performance in induced _k_OA-like changes, thus preventing the loss of chondrocytes.

## Introduction

Chondrocytes are responsible for tissue maintenance which impact on joint function and performance (Sophia Fox et al., 2009; Akkiraju and Nohe, 2015; Charlier et al., 2016). Current literature has reported exercise-induced chondroprotection in knee osteoarthritis (_k_OA) (Loeser, 2010; Golightly et al., 2012; Geneen et al., 2017). A potential explanation is the mechanical signal transduction (O’Hara et al., 1990; Urban, 1994), preserving the cartilage proteoglycans, and promoting chondrocytes modulation (Little and Ghosh, 1997; Loeser et al., 2012). Low-magnitude mechanical stress seems to suppress the pathway of interleukin-1 beta (IL1β) and tumor necrosis factor (TNF) release (Roman-Blas et al., 2009; Leong et al., 2010; Li et al., 2013; Assis et al., 2016). The IL1β and TNF are inflammatory mediators involved in joint degeneration caused by _k_OA (Blasioli and Kaplan, 2013; Mabey and Honsawek, 2015). The modulation of these cytokines in the joint would regulate the synthesis of proteoglycans and collagen, thereby attenuating the swelling process (Knobloch et al., 2008; Iijima et al., 2015).

The literature points out redox imbalance and the related increase on reactive oxygen species (ROS), swelling process and necrosis of chondrocytes as the pathophysiology of _k_OA (Altindag et al., 2007; Rose et al., 2012; Hui et al., 2016). Moreover, redox imbalance and the increased inflammatory biomarkers may cause cartilage damage, neuroinflammatory disease progression, and joint disability (Kim et al., 2010; Watari et al., 2011; Attur et al., 2013, 2015; Reed et al., 2014).

Several interventions are used to improve motor performance in patients with **_k_OA** (Golightly et al., 2012; Geneen et al., 2017), and a low-cost aerobic training may be an alternative (Hunter and Eckstein, 2009; Semanik et al., 2012). Aerobic training improves blood soluble TNF receptors level and brain-derived neurotrophic factor (BDNF) plasma level in people with _k_OA (Gomes et al., 2012; Simão et al., 2014). Moreover, BDNF seems to impact on chondrocyte differentiation, changing it from proliferative to differentiation program (Hutchison, 2012).

Beyond insufficient information about the modulatory effect of the aerobic training in the knee joint degeneration parameters and possible relationships between joint parameters and motor performance, the literature still presents gaps regarding the effect of aerobic training in the joint preservation on _k_OA-like changes. Many studies detail mechanotransduction mechanisms but still remain questions about joint function. Moreover, few studies address joint function tests to establish a new therapeutic approach in clinical practice. The current study aimed to investigate the effects of moderate-intensity aerobic training in the inflammatory and redox biomarkers modulation of _k_OA-like changes, and its possible link to motor performance in rats. Therefore, because experimental _k_OA induces joint swelling process, we hypothesized that moderate-intensity aerobic training would attenuate the swelling process, favoring the redox balance and preserving chondrocytes in rats with _k_OA -like changes. Improved modulation in joint biomarkers levels might explain an effect on motor performance.

## Materials and Methods

Male Wistar rats were used in the current study that was part of a Masters in Physiology at the Universidade Federal dos Vales do Jequitinhonha e Mucuri (Martins, 2017). The project was approved by the local Ethics Committee (protocol 005/2015).

Twenty-seven rats were randomized into three groups: sham group (SHAM), *n* = 9; induced _k_OA group (OA), *n* = 9; and induced _k_OA + aerobic training (OAE), *n* = 9. The rats had available water and food (i.e., Nuvilab CR1, Nuvital Nutrientes S/A, Brazil) as they desire, and their environment was controlled (i.e., humidity of 60% and temperature of 22°C).

### Induced Osteoarthritis

Twelve-week-old male Wistar rats were anesthetized with ketamine (80 mg/kg, i.p.) and xylazine (15 mg/kg, i.p.). Then, _k_OA was induced on the right knee joint at 90° flexion by direct infiltration of monosodium iodoacetate (MIA) (1.2 mg diluted in 50 μL saline solution). We used a 29G X 1/2 BD Ultra-FineTM insulin needle (Guzman et al., 2003; Cifuentes et al., 2010; Takahashi et al., 2018). The SHAM group received an infiltration containing 50 μL of saline solution (0.9% NaCl).

### Aerobic Training

To perform the aerobic training, a motorized treadmill (Insight^®^, SP, Ribeirão Preto, Brazil) with six individual lanes and with no inclination was used in the study. OAE group started the familiarization to the treadmill (10 min/day for 5 days) 24 h after the _k_OA-induction procedure. Then, 24 h after the familiarization period, initiated the training program, which consisted of treadmill running at the velocity of 16 m/min, 3 days per week during 8 weeks (Cifuentes et al., 2010), and the duration of running sessions was increased from 30 to 50 min at the fourth week. The workload of all groups was analyzed at the end of the training program during a treadmill incremental test (initial speed of 10 m min^–1^, 1% slope, with no electrical stimulus, an increase of 2 m min^–1^ every 3 min) (Balthazar et al., 2009; Primola-Gomes et al., 2009). The workload (W; J) was calculated as: W = body weight (kg) × total time to fatigue (min) × treadmill speed (m min^–1^) × sin θ (treadmill inclination) × 10 (Lacerda et al., 2006).

### Evaluation of Motor Performance – Forced Locomotion (Rotarod Test)

In the rotarod test (Scienlabor, Brazil), the rats are stimulated to walk around a circle drum surface. The rotarod test measures balance, coordination, physical performance, and motor-planning by calculating the number of falls during determined speed. The time that a given rat stays on this circle rod represents the joint function. We used a protocol adapted from Piel et al. (2014) to quantify the number of falls of rats during 3-min period keeping a fixed speed of 8 revolutions per minute (rpm) (Piel et al., 2014).

### Locomotion Test on a Footbridge

Rats locomoted on a footbridge to investigate joint function when moving a short distance. The footbridge had a length of 100 cm delimited by 3 mm thick aluminum filets. Image and time to complete a single pass during animals’ locomotion were recorded. Records were later analyzed by a blinded investigator to quantify the total time of displacement and the number of times each rat stepped out of the space between the filets (number of failures).

### Euthanasia

Animals were euthanized individually by decapitation and their right knee joints were analyzed. Approximately 12 mL of blood was collected in tubes containing ethylenediaminetetraacetic acid (EDTA), then centrifuged at 500 × *g* for 10 min and the serum aliquoted and frozen in a −80°C freezer for further analysis.

### Joint Lavage

For later analysis, the joint lavage (JAL) supernatants were stored at −80°C. Immediately after recovering JAL, we removed the joint capsule and stored it in a freezer at −80°C. Moreover, we homogenized the sample in phosphate buffer and also frozen it at −80°C for future analysis. IL1β, TNF, and interleukin-10 (IL10) knee joint biomarkers were analyzed according to the manufacturer’s instructions by ELISA kits (DuoSet, R&D Systems, United States).

### Macerated Joint Capsule

The capsule was placed into a beaker with 750 μL of cytokine extraction solution, and a tissue homogenizer (Tecnal, TE-103) was used to obtain the macerate. Then, the capsule macerate was processed at 8 rpm speed for 2 min, the volume centrifuged at 3500 xg at 4°C for 10 min and stored in a freezer. BDNF and interleukin-6 (IL6) levels were analyzed according to the manufacturer’s instructions by ELISA.

### Measurements of Redox Status

The reaction of the thiobarbituric acid with malondialdehyde (MDA) was used to determine lipid peroxidation by thiobarbituric acid reactive substances (TBARS) plasma levels (Ohkawa et al., 1979). The ferric reducing ability of plasma (FRAP), i.e., the reduction of ferric-tripyridyltriazine [Fe(III)-TPTZ] complex to ferrous-tripyridyltriazine [Fe(II)-TPTZ] (Benzie and Strain, 1996) was used to determine the total antioxidant capacity. The Bradford method using bovine serum albumin was used as a standard to determine the samples protein levels (Bradford, 1976).

### Histology

The right knee joints were placed in 4% neutral-buffered formalin for 24 h. After that, tissues were placed in 10% EDTA at pH 7.4 for decalcification (Jimson et al., 2014). Sagittal sections were prepared from knee joints. Two slides of the femur compromised by MIA induction or SHAM were prepared for histological analyses. Three cuts in each slide. Tissues were placed in formalin, dehydrated in a graded series of ethanol and xylol, embedded in paraffin, cut into 6 μm serial sections, and stained with hematoxylin-eosin.

### Quantification of Chondrocytes

To investigate the number of active cells, the middle third of the joint was used. For cell counting, the nuclei stained by hematoxylin present in the superficial, intermediate and transitional areas were considered. The analysis was performed on the articular facet of the right femur. The image was captured by a microscope with a 40 × magnification. The software Image J was used for cell counting. Two micrographs were taken in series and a blinded investigator analyzed them on different days (ICC = 0.99). For statistical analysis, the average number of cells was established and used.

### Statistical Analysis

We used the SPSS statistical package, version 22.0 (Inc., United States) and Graph Pad Prism, version 5.0 (Inc., United States). Data are expressed as mean ± standard error (S.E.M). Normality of data was assessed using the Shapiro–Wilk test. For comparisons, we used the one-way ANOVA with Tukey’s *post hoc* tests for parametric data (Body mass, IL6 and number of chondrocytes) and Kruskal-Wallis with Dunn’s *post hoc* test for non-parametric data (IL1β, TNF, IL10, TBARS, FRAP, number of falls, total time of displacement, and number of failures). Effect Size (d) was checked in G^∗^Power 3.1.9.2 program. Effect size conventions for test family (*F* tests) and one-way ANOVA: *d* = 0.10 (small); *d* = 0.25 (medium); *d* = 0.40 (large). The Spearman correlation investigated associations between two intra-articular biomarkers of joint damage (IL1β, TNF) and joint function tests: number of falls, total time of displacement and number of failures.

To determine intra-examiner reliability for the evaluated outcomes, the intraclass correlation coefficient (ICC) adopting a 95% confidence interval was determined. Multiple linear stepwise regression models confirmed the association between selected biomarkers and joint function, adjusting by Bonferroni at α = 0.017. Graphs were built using the GraphPad Prism 5 (GraphPad Software Inc., San Diego, CA, United States).

## Results

**FIGURE 1:**
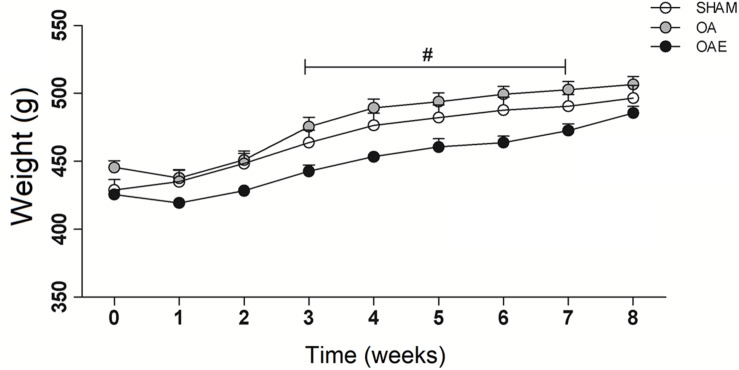
Body mass over the 8-week interventions. (White circle) SHAM group; (Gray circle) OA: knee osteoarthritis group; (Black circle) OAE: knee osteoarthritis plus aerobic training group. *N* = 9 per group. Data are reported as Mean ± S.E.M. ^#^Difference between OA vs. OAE.

Twenty-seven animals were available for this study, recovered uneventfully from surgery and exercise procedure. By the end of the study, the rats were about 6 months old. No significant differences were observed in body weight between the three groups at the surgery and at the 8th-week post-surgery (Figure 1).

**FIGURE 2:**
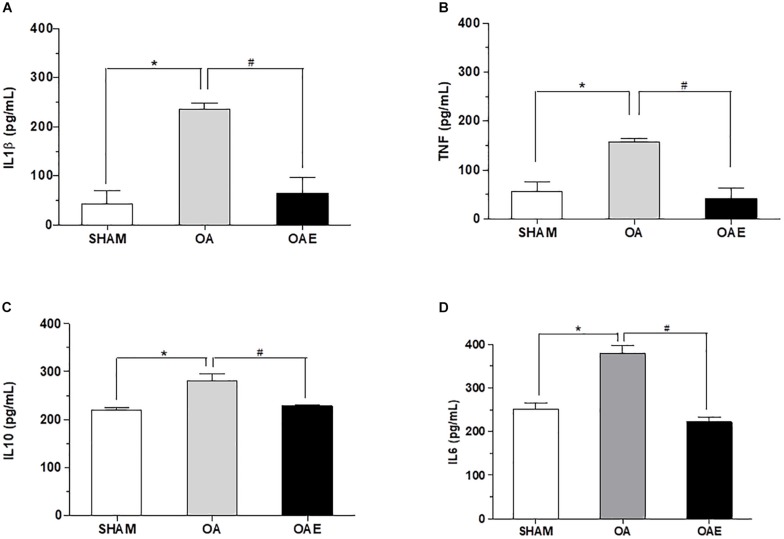
Effect of aerobic training on inflammatory biomarkers. **(A)** Interleukin-1beta (IL1β) measured in joint wash fluid; **(B)** Tumor necrose factor (TNF) measured in joint washed; **(C)** Interleukin-10 (IL10) measured in joint washed, and **(D)** Interleukin-6 (IL6) measured in the joint capsule for SHAM group; OA: knee osteoarthritis group; OAE: knee osteoarthritis plus aerobic training group. *N* = 9 per group. Data are reported as Mean ± S.E.M. ^#^Difference between OA vs. OAE. ^∗^Difference between OA vs. SHAM.

Aerobic training increased the total workload of OAE rats by 64% as compared to sham and OA groups (SHAM: 23.8 **±** 8.0 J, OA: 23.7 **±** 11.0 J, OAE: 69.2 **±** 15.9 J, *p* = 0.0002, *d* = 0.85). Levels of IL1β, TNF, IL10 in the joint washed and the level of IL6 in the joint capsule increased respectively by 5.55, 2.84, 1.27, and 1.50-fold in the OA group. In OAE group compared with OA group, the aerobic training modulated levels of these joint cytokines close to those in the SHAM group (IL1β: *p* < 0.0001, *d* = 1.18; TNF: *p* = 0.0001, *d* = 1.00; IL10: *p* < 0.0001, *d* = 1.32; IL6: *p* = 0.0001, *d* = 1.68) (Figure 2).

**FIGURE 3:**
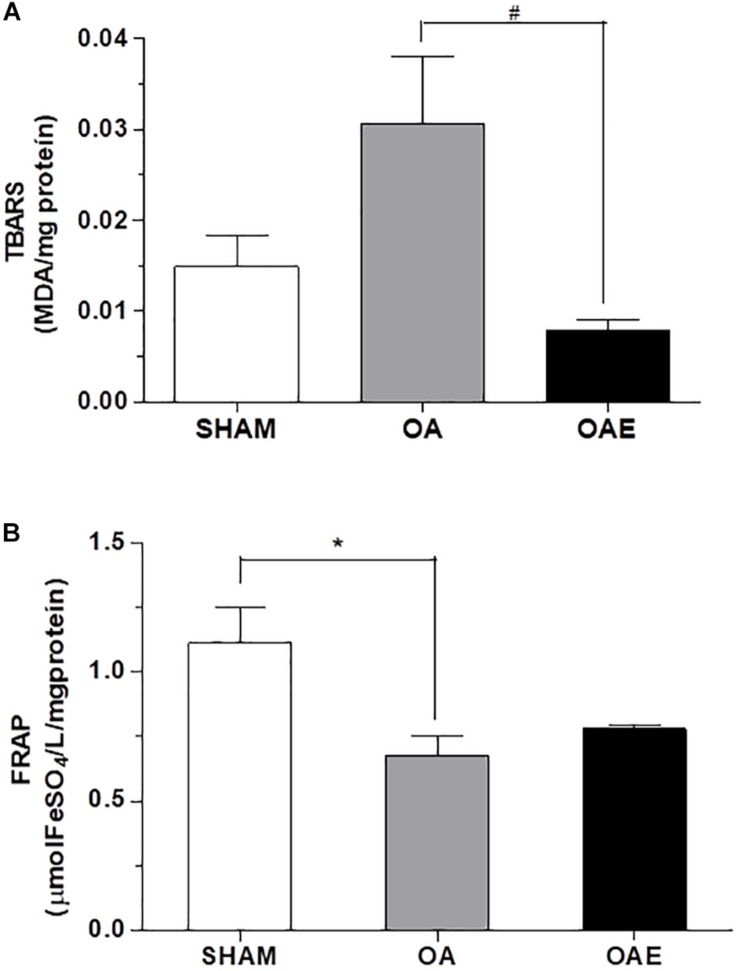
Effect of aerobic training on status redox biomarkers. **(A)** Thiobarbituric acid-reactive substance (TBARS) measured in plasma; **(B)** The ferric reducing ability of plasma (FRAP) measured in plasma for SHAM group, OA: knee osteoarthritis group; OAE: knee osteoarthritis plus aerobic training group. *N* = 9 per group. Data are reported as Mean ± S.E.M. ^#^Difference between OA vs. OAE. ^∗^Difference between OA vs. SHAM.

The BDNF level in the OAE group was 41% higher than in the OA group (*p* = 0.05; *d* = 0.86). The induction of _k_OA-like changes increased by 1.14 times the TBARS plasma level and reduced the FRAP plasma level by 6.83% (*p* = 0.02) in the OA group compared with SHAM group. In OAE group compared with OA group, the aerobic training returned the plasma level of TBARS close to those in the SHAM group, without changing the total antioxidant capacity (TBARS: *p* = 0.001; *d* = 0.86; *P* = 0.95; FRAP: *p* = 0.05; *d* = 0.62) (Figure 3).

**FIGURE 4:**
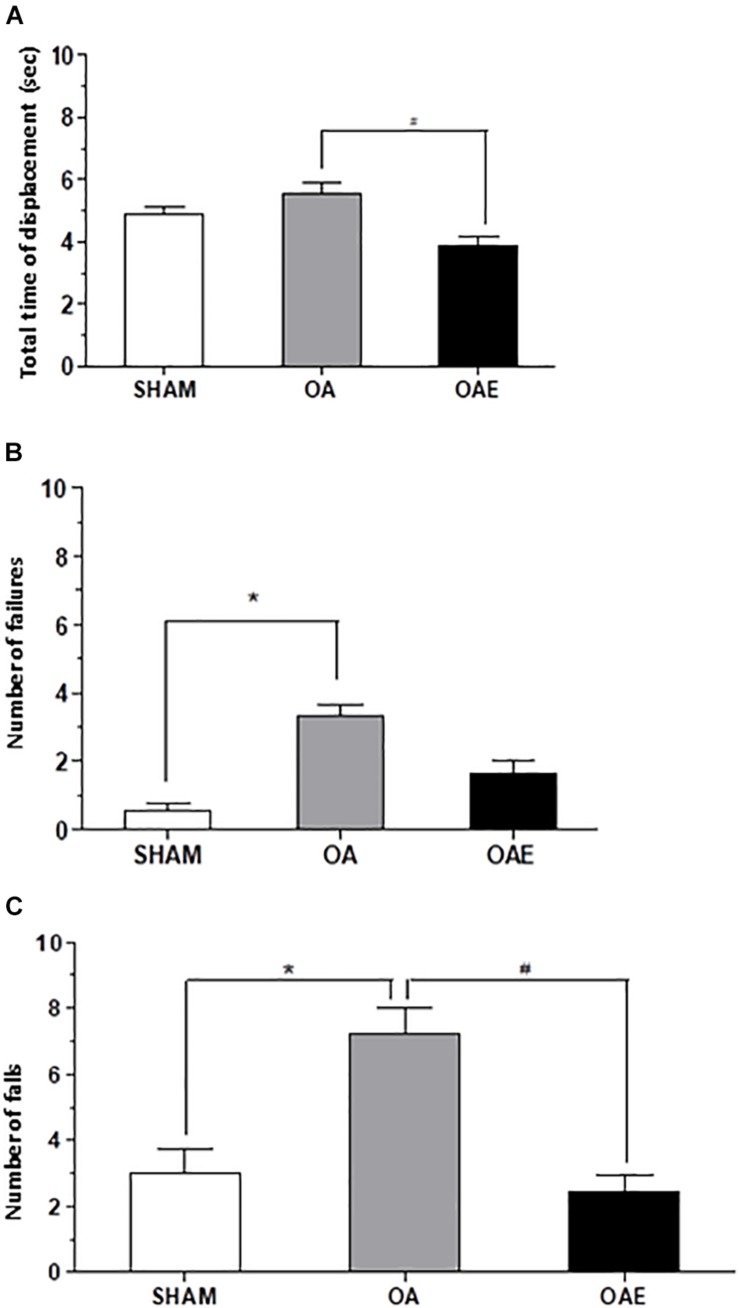
Effect of treadmill aerobic training on joint function tests. **(A)** Total time of displacement; **(B)** Number of failures, and **(C)** Number of falls in SHAM group, OA: knee osteoarthritis group; OAE: knee osteoarthritis plus aerobic training. *N* = 9 per group. Data are reported as Mean ± S.E.M. ^#^Difference between OA vs OAE; ^∗^Difference between OA vs. SHAM.

The analysis of the joint function tests showed that the _k_OA-induction (OA group) increased by 1.13 times the total time of displacement on a 100 cm footbridge and increased by 6.05 times the number of failures in the course when compared with SHAM group. The number of falls during the rotarod test increased 2.40 times in the OA group compared with SHAM. Therefore, in OAE group compared with OA group, the aerobic training had a positive effect on the disease, since it improved the performance in the three parameters evaluated, matching the SHAM score (Number of falls: *p* = 0.002; *d* = 1.05; Number of failures: *p* = 0.0002 *d* = 0.26; Total time of displacement: *p* = 0.005; *d* = 0.72) (Figure 4).

Number of falls (*rs* = 0.69; *p* = 0.0003) and number of failures (*rs* = 0.66; *p* = 0.0002) were associated with the IL1β. Functional performance measures: number of falls in the rotarod test (*rs* = 0.73; *p* < 0.0001); total time of displacement (*rs* = 0.63; *p* = 0.0005); and number of failures (*rs* = 0.52; *p* = 0.005) were associated with the TNF. Multiple linear stepwise regression models of IL1β and TNF explained 58% of the variability in the number of falls (IL1 β: *p* = 0.001; TNF: *p* = 0.002). TNF only explained 29% of the variability of the total time of displacement (*p* = 0.02) and 21% of the variability of the number of failures (*p* = 0.02). The increase of 1 pg/mL in intra-articular IL1β level leads to an increase of 0.40 points in the number of falls. The increase of 1 pg/mL in the intra-articular TNF level leads to an increase of 0.51 points in the number of falls; 0.57 s in the total time of displacement; and 0.49 points in the number of failures (Table 1).

**TABLE 1:** Multiple linear stepwise regression analysis.

**Joint damage biomarkers**		**Number of falls**		**Total time of displacement**				**Number of failures**	
			
	**B**	**p**	**R^2^**	**β**	**P**	**R^2^**	**B**	****p****	**R^2^**
			0.58			0.29			0.21
IL1β (pg/mL)	0.40	0.001^∗^		−0.003	0.99		0.32	0.10	
TNF (pg/mL)	0.51	0.002^∗^		0.57	0.02^∗^		0.49	0.02^∗^	

*β, Standardized regression coefficient; *R*^2^, Adjusted R square. IL1β, Interleukin-1 beta; TNF, Tumor necrosis factor.*

**FIGURE 5:**
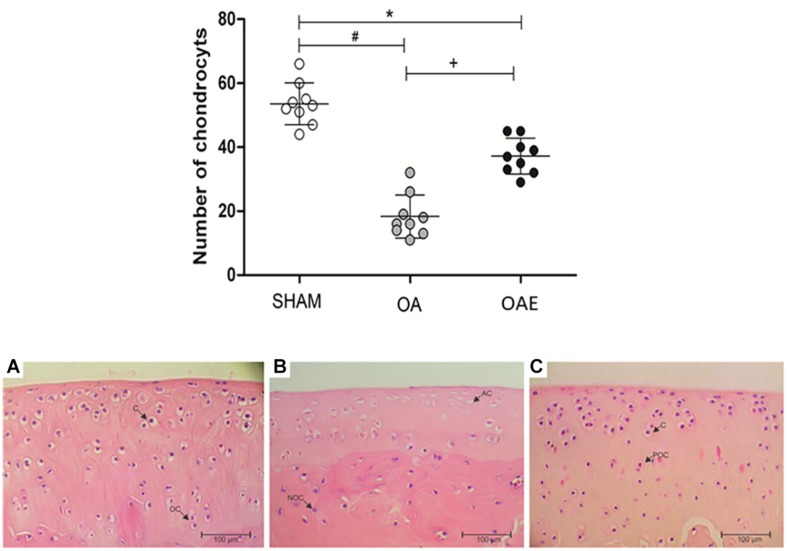
Effect of moderate-intensity aerobic training on the number of chondrocytes, and representation of the histological cell number (chondrocytes). SHAM group (SHAM): white circles and **A**; knee osteoarthritis group (OA): gray circles and **B**; knee osteoarthritis plus moderate-intensity aerobic training group (OAE): black circles and **C**. *N* = 9 per group. Data are reported as Mean ± S.E.M. ^+^Difference between OA vs. OAE; ^∗^Difference between OAE vs. SHAM. ^#^Difference between OA vs. SHAM. **C:** chondrocytes; OC: organized cells; AC: the absence of cells; NOC: no organized cells; POC: partially organized cells.

Quantification of hematoxylin-stained nuclei showed that the induction of _k_OA reduced the number of cells (chondrocytes) alive by approximately 35% compared with the SHAM group. Aerobic training (OAE group) prevented chondrocyte cell death since the number of active cells in the trained group was 51.35% higher than in the OA group (*p* < 0.0001) (Figure 5).

## Discussion

Overall, the current study showed an association between worse joint function and high levels of joint degeneration biomarkers, as indicated by high levels of TNF and IL1β. Moreover, aerobic training could reverse local inflammatory biomarkers and decrease systemic MDA level with an improvement in gait tasks, motor, and physical performance. Histological data of the femoral joint also confirmed the beneficial effect of the proposed exercise to the _k_OA-induced.

Scientific evidence suggests the involvement of inflammatory biomarkers and redox status parameters for the advancement and progression of _k_OA (Regan et al., 2005; Koike et al., 2015). In patients with _k_OA, chondrocytes and synovial cells stimulate the production of inflammatory cytokines, i.e., IL1β and TNF (Altindag et al., 2007; Kim et al., 2010; Wojdasiewicz et al., 2014; Kunisch et al., 2016). Kim et al. (2010) evidenced that IL1β and TNF are responsible for mitochondrial DNA damage in _k_OA, promoting the development of ROS and chondrocyte death (Kim et al., 2010).

The compression forces of low magnitude during physical exercise seem to promote physiological control. Such control modulates the synthesis of collagen and proteoglycans possibly inhibited in swelling joints (Quinn et al., 1998; Fehrenbacher et al., 2003; Park et al., 2004). This could indicate the greater maintenance of proteoglycan in the joint cartilage of rats exposed to moderate exercise (Galois et al., 2003; Cifuentes et al., 2010; Li et al., 2013; Mohammadi et al., 2013). Thus, the authors suggest that the positive effects are linked to the decrease of chondrocyte necrosis in the experimental group, lowering metabolites of cell death and induction of inflammatory factors expression (Galois et al., 2003). The high level of ROS would act on the expression of cytokines, making worse the swelling. The ROS may initiate the cartilage degeneration and advancement of lipid peroxidation in chondrocytes (Yudoh et al., 2005; Ostalowska et al., 2006; Kim et al., 2010; Watari et al., 2011).

In _k_OA, inflammatory cytokines alter the joint biochemical balance inducing chondrocyte necrosis. The production and secretion of catabolic cytokines augment ROS production inducing a redox imbalance. Free radicals interacting with chondrocyte DNA acts directly by altering cellular components, i.e., proteoglycans, collagens, and protein oxidation, favoring cartilage degeneration, which may compromise the thickness of the synovial fluid, and the synthesis of other components (Henrotin et al., 2003; Rose et al., 2012; Reed et al., 2014). Physical and functional damages occur due to tissue structural change. Catabolic cytokines (IL1β and TNF), and regulatory cytokine (IL6) are probably the main factors in this process, facilitating paths of degeneration by activating paths such as matrix metalloproteases (Rojas-Ortega et al., 2015; Assis et al., 2016). The augmented inflammatory profile marks the loss of extracellular matrix integrity, developing an oxidative injury, and, lastly, the chondrocytes death. The death of these chondrocytes seems related to the compromised joint function since this cellular type is responsible for the mobilization of essential components that assure the main functions of load distribution and reduction of friction during static or dynamic exercises that guarantee the joint function. Mohammadi et al. (2013) assessed histological data of depth ratio of lesions demonstrating that 4 weeks of moderate exercise almost treated _k_OA symptoms in rats (Mohammadi et al., 2013). Moreover, the level of MDA increased in induced _k_OA dogs. This intensification revealed degeneration of the type II collagen (Goranov, 2007), implying a relationship between redox imbalance and cartilage degeneration. Oxidative injury can result in cell death, triggering particles and oxidized molecules release, cellular degeneration, and increased inflammation.

_k_OA subjects have greater ROS plasma level and lower antioxidant supplies (Abruzzo et al., 2013; Germanou et al., 2013). The redox imbalance can play a critical role in the cartilage degeneration (Henrotin et al., 2003; Reed et al., 2014). Thus, in both human and animal, _k_OA transporters have a high level of systemic biomarkers which means cellular damage led by ROS. As a consequence, cell-matrix may be compromised. Inhibition of this course can successfully avoid degeneration of articular cartilage and neo-formation of type II collagen (Poole et al., 2002).

The transduction of mechanical signals of dynamic pressure in chondrocytes may favor the pathways that counteract tissue catabolism. Li et al. (2013) assessed anabolic responses on bovine cartilage *in vitro* cells culture inducing matrix biosynthesis with different compression ranges (10, 20, and 30%) and demonstrated that moderate dynamic compression can exert an “anti-catabolic” effect, and suppress the expression of TNF, IL6, and soluble IL6 receptors (Li et al., 2013). The catabolism control appears to relate to a range of compression frequency, amplitude, and to the low-to-moderate intensity load, showing the importance of a voltage amplitude threshold for the regulation of inflammatory paths and cell survival (Cifuentes et al., 2010; Beckett et al., 2012; Rojas-Ortega et al., 2015; Rios et al., 2018). Moreover, the exercise load influences BDNF production and release (Nofuji et al., 2008; Yarrow et al., 2010; Szuhany et al., 2015). BDNF is an important growth factor expressed in joint chondrocytes and in epiphyseal plaques of _k_OA subjects. BDNF causes the growth and, mainly, differentiation (Hutchison, 2012) of chondrocytes, inducing the proliferation pathway that can act as a restoration mediator. Furthermore, BDNF level has been also systemically increased in patients with _k_OA (Simão et al., 2014).

Because mild- to moderate-intensity exercises seem to play an anti-inflammatory role, we decided to perform a moderate-intensity aerobic training on a treadmill to promote mechanical biostimulation caused by joint compression (Galois et al., 2003; Cifuentes et al., 2010; Li et al., 2013; Assis et al., 2016; Rios et al., 2018). In the current study, we decided to use an aerobic training similar to the study of Cifuentes et al. (2010) that investigated the effects of impact exercise on the _k_OA-induced cartilage aspects in rats. This protocol was chosen because the data of that investigation demonstrated that aerobic training contributed to the preservation of some joint cartilage parameters in experimental _k_OA. Moreover, the overload to the intensity and inclination of exercise sessions were not applied, once it could directly affect the dynamic compression of knee joint cartilage cells, leading to misinterpretations of our results. Thus, active mechanotransduction induces changes in oxygen tension and subsequent positive effects on matrix synthesis and cell growth (Urban, 1994; Park et al., 2004). These effects are explained by the displacement of growth factors or cellular cytokines by shifting cellular metabolism (Tilwani et al., 2017). Finally, our results showed that the proposed training offered an inflammatory control, confirmed by the modulation effect in biomarkers levels (IL1β and TNF in the joint wash; IL10 and IL6 in the knee joint).

The positive effects of aerobic training are attributed to the ability of the suppression of signals transduction paths of inflammatory and catabolic mediators together with the stimulation of anabolic paths. *In vitro* studies have verified that mild to moderate mechanical stress inhibits swelling by suppression of IL1β, TNF and the transcription of various joint degeneration inflammatory biomarkers (Fehrenbacher et al., 2003; Rose et al., 2012; Yamabe et al., 2013). Experimental _k_OA studies showed a positive effect of aerobic training, whereas high-intensity training had a deleterious effect (Beckett et al., 2012; Ni et al., 2012; Rojas-Ortega et al., 2015; Li et al., 2017). These data seem to determine the role of aerobic exercise appropriate dose (intensity, frequency, and duration) in modulating chondrocyte response (Ni et al., 2013; Na et al., 2014; Hill et al., 2017). In rats without previous _k_OA induction, high-intensity exercises in many treadmill angles were not able to induce knee damage (Beckett et al., 2012; Rios et al., 2018). Thus, biochemical responses appear to be sensitive to the force only in the injury. Our data once again is in accordance to this premise since the proposed protocol was effective in dropping the joint IL1β and TNF in the trained group, as well as in reducing the TBARS systemic level, augmenting knee joint function and physical performance. In the present study, we identified an increased chondrocytes number in the OAE group. It is already known that chondrocytes are responsible for tissue maintenance which impact on joint function and motor performance (Sophia Fox et al., 2009; Akkiraju and Nohe, 2015). Thus, we speculate that the largest number of chondrocytes and the lower joint degradation cytokines level (IL1β and TNF) (Kim et al., 2010; Li et al., 2013, 2015; Rojas-Ortega et al., 2015) might have preserved the proteoglycans and collagen joint which lead to better motor performance of the OAE group compared to the OA group. Based on the results, exercise prevented the increase of inflammatory biomarkers, and, consequently, prevented the loss of chondrocytes (the only marker evaluated). We cannot extrapolate our results to the whole joint, because the number of chondrocytes was the only analysis. This theory needs to be better clarified ahead. Our study is innovative as it points out the benefits of a therapeutic approach to an experimental OA model. Assessing the effect on physical performance of the animals it was demonstrated that the dose-controlled impact is achieved by ways not fully elucidated in this study. The modulation of inflammation, due to the known effects of aerobic training, has affected the redox status balance, lowering oxidative damage, improving motor performance in functional tasks.

A limitation of this study was that we did not determine _k_OA-like changes according to the OARSI score. However, it was not possible because we used a single hematoxylin and eosin (H&E) staining for analysis. Thus, immunohistochemistry assessments on cartilage tissue would be useful in future studies.

## Conclusion

To conclude, a moderate-intensity aerobic treadmill training appears to modulate chondrocytes via activation of anabolic paths, swelling control by IL1β and TNF levels modulation, systemic TBARS level lowering and positive regulation in joint BDNF level, resulting in physical and motor performance improvements. As a perspective, the efficacy of the training protocol used here should be investigated in older animals.

## Data Availability

The datasets generated for this study are available on request to the corresponding author.

## Ethics Statement

This animal study was reviewed and approved by the Commission on Ethics in Animal Use of the Universidade Federal dos Vales do Jequitinhonha e Mucuri (protocol 005/2015). Written informed consent was obtained from the owners for the participation of their animals in this study.

## Author Contributions

JM, VM, MO, HL, AC, CC, and AL conceived and designed the study. JM, VM, GA, SF, JS, RT-G, DS, MO, HL, AC, AF, CC, JP, and AL contributed to analysis and interpretation of the data. JM, VM, SF, SS, JS, RT-G, DS, TD, and AL drafted the article. JM, VM, SF, MO, HL, AC, AF, CC, JP, MB-F, and AL critically revised the article for important intellectual content. JM, VM, GA, SF, JS, RT-G, DS, MO, HL, AC, AF, CC, JP, VO, MB-F, and AL approved the final article. JM, SF, MO, HL, AC, JP, and AL statistically expertised the study. VM, MO, HL, AC, AF, CC, and AL provisioned the study materials. VM, GA, RT-G, DS, MO, AF, CC, and AL contributed to administrative, technical, or logistic support.

## Conflict of Interest Statement

The authors declare that the research was conducted in the absence of any commercial or financial relationships that could be construed as a potential conflict of interest.
